# High expression of AGR2 in lung cancer is predictive of poor survival

**DOI:** 10.1186/s12885-015-1658-2

**Published:** 2015-10-06

**Authors:** Mohammed Alavi, Vei Mah, Erin L. Maresh, Lora Bagryanova, Steve Horvath, David Chia, Lee Goodglick, Alvin Y. Liu

**Affiliations:** 1 Department of Pathology and Laboratory Medicine, David Geffen School of Medicine, University of California, Los Angeles, 10833 Le Conte Ave., Los Angeles, CA 90095 USA; 2 Department of Statistics, David Geffen School of Medicine, University of California, Los Angeles, 10833 Le Conte Ave., Los Angeles, CA 90095 USA; 3 Jonsson Comprehensive Cancer Center, David Geffen School of Medicine, University of California, Los Angeles, 10833 Le Conte Ave., Los Angeles, CA 90095 USA; 4 Department of Urology, University of Washington, 1959 NE Pacific St., Seattle, WA 98195 USA; 5 Institute for Stem Cell and Regenerative Medicine, University of Washington, 850 Republican St., Seattle, WA 98195 USA

**Keywords:** Lung cancer, AGR2, Survival

## Abstract

**Background:**

Anterior gradient 2 (AGR2) is a protein disulfide isomerase-like protein widely expressed in many normal tissues as well as cancers. In our study, non-neoplastic bronchial epithelial cells as well as non-small cell lung cancer (NSCLC) cells express AGR2 protein.

**Methods:**

AGR2 expression was analyzed on lung tissue microarrays. Tumor staining was correlated with clinical outcomes.

**Results:**

On a lung cancer tissue microarray using immunohistochemistry, expression levels in cancer showed generally decreasing intensities in order from adenocarcinomas with mucinous components, other adenocarcinomas, squamous carcinomas, to large cell carcinomas. The study cohort was comprised of 400 cases. As a group, there was a slight trend of lower expression with increasing tumor grade. AGR2 expression level was a significant predictor of overall survival in younger patients only. Patients under 65 with lower levels showed a significantly better survival for both men and women. Patients over 65, in contrast, showed no such trend.

**Conclusions:**

Nearly all NSCLC tumors show AGR2 expression. Lung cancer expression of AGR2 has prognostic value for younger patients.

## Background

Lung cancer is the leading cause of cancer related deaths in the United States and many parts of the world. According to the American Cancer Society approximately 224,210 individuals in the United States will be diagnosed with the disease and 159,260 (71 %) will die of it in 2014 [1]. Most of these are classified as non-small cell lung cancers (NSCLC), and include adenocarcinomas, squamous carcinomas, large cell carcinomas as well as less commonly occurring histopathologic patterns such as adenosquamous carcinoma. The search continues for bio-markers which can delineate outcomes related to long term survival or response to treatments.

Anterior gradient 2 (AGR2) is expressed by many solid tumor types and is known as an adenocarcinoma antigen [2]. In prostate cancer, expression of AGR2 is increased in the tumor epithelial cells; normal luminal cells have no detectable expression [3, 4]. In bladder cancer, expression of AGR2 is absent in ~75 % cases; normal urothelial cells have moderate AGR2 expression (in comparison to that in prostate cancer cells) [5]. Expression in primary prostate cancer is associated with lower grade disease (and longer biochemical recurrence-free survival) [6, 7]. Breast cancer also shows AGR2 expression [8]. Like prostate cancer, high AGR2 expression is associated with low grade and low expression with high grade. Besides differential expression in tumor grades, AGR2 expression is found preferentially in the adenocarcinoma type than the squamous cell carcinoma type of NSCLC [9]. In esophageal cancer, AGR2 can be used to improve distinguishing adenocarcinoma (99 % positive) from squamous cell carcinoma (37 % positive, typically in focal areas) in some circumstances [10]. However, oral squamous cell carcinoma has recently been reported to express AGR2 [11].

From the foregoing description, it would appear that cancer expression of AGR2 is more associated with diseases that have a lower potential of progression. This contrasts with the molecular functioning of AGR2 in cancer cells. Cell lines transfected with AGR2 produced metastasis in a xenograft model [12], showed gain of anchorage-independent growth and promoted tumor growth [13]. In gastric cancer cells, AGR2 expression was found up-regulated in a cancer cell subline with high metastatic potential for invasion to lymph nodes [14]. Although AGR2 positive primary prostate tumors were associated with better clinical outcome than AGR2 negative tumors, many distal prostate cancer metastases in bone and visceral organs showed strong immunostaining for AGR2 [7]. In ovarian cancer, high AGR2 expression was reported in 12 % high-grade serous type, which showed significantly lower overall survival [15]. Thus, AGR2 has a prognostic value for some cancers, but this is variable.

The widespread expression of AGR2 in human cancer underscores its potential importance in tumor biology. In some organs like the prostate and pancreas, AGR2 is absent in normal epithelial cells and present in cancer cells, while in others like the bladder, AGR2 is present in normal urothelial cells but absent in cancer cells of a majority of tumors. AGR2 expression being a feature of low grade cancer appears to contradict the metastasis promoting property of AGR2. This may be due more to our lack of understanding about the precise molecular functioning of AGR2 in cancer. In this paper, we describe AGR2 expression in NSCLC and adjacent non-neoplastic lung tissue. We also show it has prognostic value in younger NSCLC patients.

## Methods

### Ethics statement

This research was approved by the UCLA Office of Human Research Protection Program (IRB 11–00–1301) and the UW-Fred Hutchinson Cancer Research Center Institutional Review Board (Protocol 9147). For use of pre-constructed lung tissue microarrays (TMA) at UCLA, this study did not meet the definition of human subjects research, so no patient signatures were obtained again as they were not required. No children participants were enrolled.

### Lung tissue microarrays

The lung TMA was constructed with archival formalin-fixed, paraffin embedded lung tissue samples and has previously been described in detail [16, 17]. Tissues sampled included primary lung tumor, as well as adjacent non-neoplastic lung parenchyma and metastatic lung carcinoma to lymph nodes and distant sites. All tumors were reviewed by at least two pathologists to confirm the diagnoses. At least three core tissue biopsies, 0.6 mm in diameter, were taken from morphologically representative regions for all histologies sampled and precisely arrayed using a custom built instrument as described before [16]. The TMA contained 1,202 informative tissue cores of NSCLC types and 132 cores of non-neoplastic bronchial/bronchiolar tissue from a cohort of 400 patients. The NSCLC types included adenocarcinoma, squamous carcinoma and large cell carcinoma as well as smaller numbers of neuroendocrine (carcinoid and atypical carcinoid) and adenosquamous carcinomas. A detail of the patient demographics is shown in Table 1.

**Table 1:** Patient demographics and AGR2 expression by subgroups

AGR2 expression (integrated mean intensity) by subgroups			
Group	n	(mean ± SEM)	*P*-value
Gender			0.025^b^
Women	205	1.33 ± 0.04	
Men	194	1.18 ± 0.04	
Stage			0.202^a^
Stage I	224	1.29 ± 0.04	
Stage II	70	1.16 ± 0.07	
Stage III	77	1.30 ± 0.07	
Stage IV	27	1.09 ± 0.09	
Grade			0.004^a^
Grade 1	63	1.34 ± 0.08	
Grade 2	112	1.33 ± 0.06	
Grade 3	158	1.26 ± 0.05	
Grade 4	35	0.94 ± 0.07	
Histology			1.07e-11^a^
Adenocarcinoma	233	1.43 ± 0.04	
Adenosquamous carcinoma	19	1.36 ± 0.14	
Squamous cell carcinoma	104	1.00 ± 0.05	
Undifferentiated carcinoma	33	0.96 ± 0.07	
Smoking status			3.50e-04^a^
Non-smokers	52	1.55 ± 0.09	
Former smokers	158	1.27 ± 0.04	
Current smokers	128	1.12 ± 0.05	

^a^Kruskal-Wallis test

^b^Mann-Whitney U test

### TMA immunohistochemistry

The lung TMA was stained using a standard two-step indirect immunohistochemistry similar to previous experiments described [6]. Briefly, tissue sections were cut immediately prior to being stained, deparaffinized in xylene, and rehydrated in graded ethanol solutions. Endogenous peroxidase was quenched with 3 % hydrogen peroxide. The sections were placed in a 95 °C solution of 0.01 M sodium citrate buffer pH 6.0 for antigen retrieval.

AGR2 expression was evaluated on the TMA with NB110-17780 rabbit polyclonal (Novus Biologicals) at 2 μg/ml concentration as described [6]. Specific staining was detected by applying anti-rabbit horseradish peroxidase-conjugated secondary antibody and Vector ABC (Vector Laboratories, Burlingame, CA). As negative control, staining was performed with concentration-matched non-immune IgG. This antibody was used extensively in our prostate and bladder cancer immunostaining study with good performance.

Semiquantitative assessment of staining was performed by a pathologist (MA) without prior knowledge of clinical information. A number value was calculated based on staining intensity (0 = not detectable, 1 = weak, 2 = moderate, 3 = strong) and percentage of cells staining at each intensity level (0–100 %). A final integrated value of intensity and frequency was determined using the formula: [(3x) + (2y) + (1z)]/100, where x, y, and z were the percent staining at intensity levels 3, 2, and 1, respectively. For outcomes analysis based on tumor expression of the marker, a pooled value for each patient was determined by taking the mean expression across cores with the appropriate tumor histology. We have used this study methodology previously [6, 7, 16–25].

### Statistical analysis

Lung cancer AGR2 expression was examined for correlations with tumor characteristics and overall survival. Briefly, the non-parametric Mann–Whitney and Kruskal-Wallis tests were used for two-group and multi-group comparisons. Correlations of continuous or ordered variables were calculated using Spearman Correlation. Patients were dichotomized at the median level of AGR2 expression for survival differences and visualization was by Kaplan-Meier plots. The Cox proportional hazards model was used to test the statistical significance of predictors in both a univariate and a multivariate setting. Logrank *P* values were used and *P* < 0.05 was considered significant. All statistical analyses were performed with the freely available R software (https://www.r-project.org/).

## Results

### Expression patterns of AGR2

**Fig. 1:**
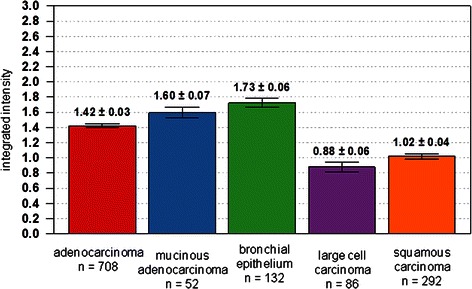
AGR2 staining intensity of lung tissue. Staining is scored by integrated intensity values on the vertical axis for the tissue types listed on the horizontal axis. Normal bronchial epithelium shows the strongest staining for AGR2. The number of tissue examples scored is indicated by n

**Fig. 2:**
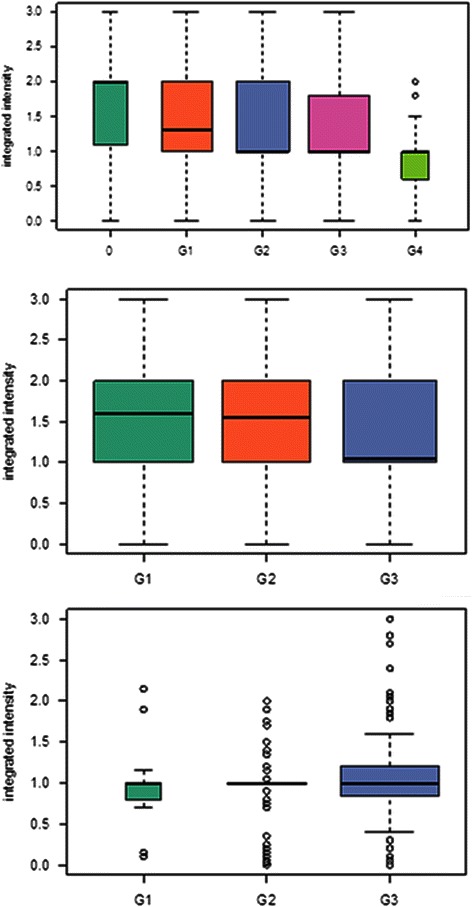
AGR2 expression and tumor grade. The box plots show a decrease in integrated intensity values with increasing tumor grades (top panel). This correlation was not evident when separated by tumor types (middle panel for adenocarcinoma; bottom panel for squamous carcinoma). Adenocarcinoma grade 3 shows low staining intensity but squamous carcinoma shows higher staining intensity

**Fig. 3:**
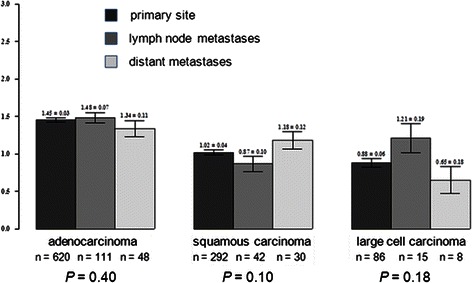
AGR2 expression in primary *vs*. metastatic sites. No significant differences in AGR2 expression was seen in primary, lymph node metastasis, and distant metastasis. This applied to adenocarcinomas, squamous carcinomas and large cell carcinomas

**Fig. 4:**
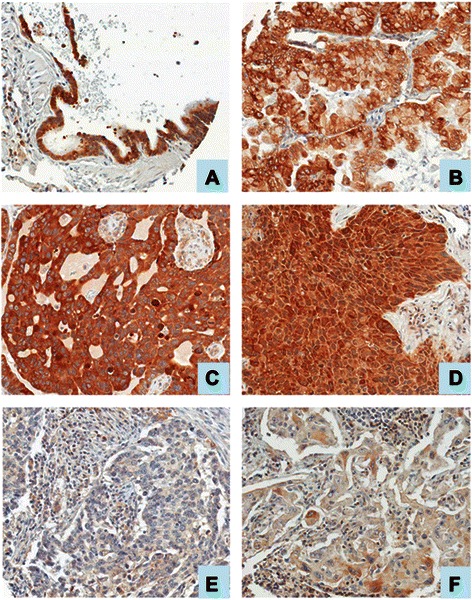
AGR2 immunostaining of lung epithelial cells. Shown are examples of lung epithelial cells stained for AGR2: **a**, normal bronchiolar epithelium; **b**, bronchioloalveolar carcinoma, mucinous type; **c**, moderately differentiated adenocarcinoma; **d**, moderately differentiated squamous carcinoma; **e**, poorly differentiated adenocarcinoma; **f**, large cell carcinoma

Higher levels of AGR2 expression were seen in non-neoplastic bronchial epithelium compared to NSCLC cells. This is illustrated in Fig. 1, which shows comparisons at the individual tissue core level. For cancer cells, adenocarcinomas displayed slightly stronger staining on average than squamous or large cell carcinomas. Of the 400 patient cases, only 7 had tumors with no staining comprising 1 % of adenocarcinoma, 4 % of squamous carcinoma, and 4 % of large cell carcinoma. Although in all NSCLC, AGR2 expression appeared to be inversely correlated to grade (*P* = 0.004), the major contributor was the lower expression in grade 4 tumors (large cell carcinomas). When examining adenocarcinomas and squamous carcinomas separately the grade correlation was absent (Fig. 2). No significant differences were seen in tissue staining in primary sites, lymph node metastases and distant metastases (Fig. 3). Examples of cell staining are shown in Fig. 4. AGR2 was predominantly localized to the cytoplasm, although occasionally there was sparse and faint nuclear staining.

**Fig. 5:**
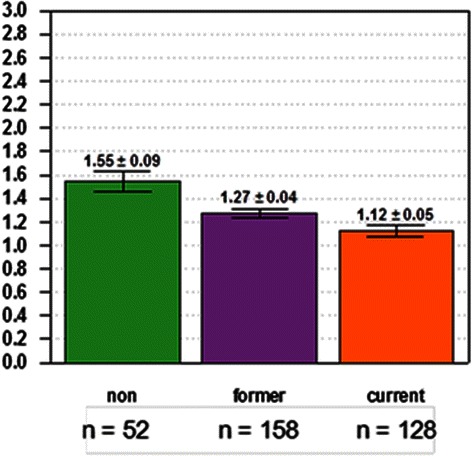
AGR2 expression and smoking status. Non-smokers had on average slightly higher levels than smokers

Tumor AGR2 expression was arbitrarily sorted into high with integrated intensity >1, and low with integrated intensity ≤1. Tables 2 and 3 shows the percentages of high and low AGR2 expressors among the different grades. For all tumor cores, the high AGR2 fractions were 59 % for grade 1, 48 % for grade 2, 43 % for grade 3, and 22 % for grade 4. This reflected the overall decrease in AGR2 staining with increasing grade. When segregated by tumor types, high AGR2 expression was more pronounced in adenocarcinoma: 62 % for grade 1, 62 % for grade 2, and 51 % for grade 3; whereas in squamous carcinoma: 19 % for grade 1, 20 % for grade 2, and 31 % for grade 3. Comparisons with other clinical variables were also examined. Interestingly, there were differences in expression by smoking status. Non-smokers had the highest levels, followed by former smokers and current smokers (*P* = 3.5E-04, Fig. 5). Current smokers included those smoking at the time of diagnosis as well as those who had quit within a year. Overall expression was slightly higher in women than men (*P* = 0.025). There was no correlation between stage and AGR2 expression (*P* = 0.21, ρ = −0.06). A summary of the findings is shown in Table 1.

**Table 2:** High *vs*. low tumor AGR2 expression. The percentages of tissue cores showing low *vs*. high integrated intensity values for the different tumor grades in adenocarcinoma and squamous carcinoma are tabulated

All tissue cores					
Grade	0	G1	G2	G3	G4
Low AGR2 (%)	24	41	52	57	78
High AGR2 (%)	76	59	48	43	22

**Table 3:** High *vs*. low tumor AGR2 expression. The percentages of tissue cores showing low *vs*. high integrated intensity values for the different tumor grades in adenocarcinoma and squamous carcinoma are tabulated

Adenocarcinoma			
Grade	G1	G2	G3
Low AGR2 (%)	38	38	49
High AGR2 (%)	62	62	51
Squamous carcinoma			
Grade	G1	G2	G3
Low AGR2 (%)	81	80	69
High AGR2 (%)	19	20	31

### Tumor AGR2 expression and overall patient survival

**Fig. 6:**
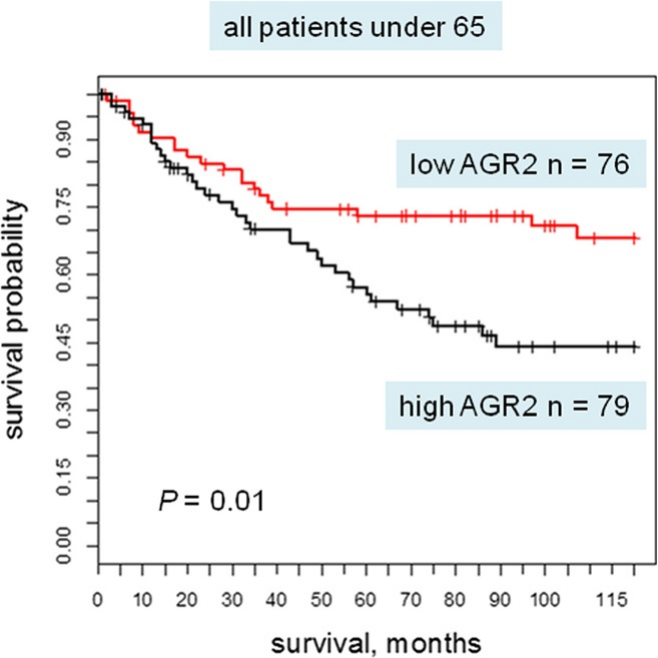
Kaplan-Meier survival plot for AGR2 expression levels. For patients under 65, high tumor AGR2 expression is correlated with a poorer survival than low tumor AGR2 expression

The entire cohort was examined at first for correlation between AGR2 and overall survival. AGR2 as a predictor did not quite reach significance, with the univariate Cox proportional hazards model *P* = 0.077 and hazard ratio (HR) = 1.2. The population was then divided by gender, age, smoking status and histology. With the univariate Cox proportional hazards model, AGR2 was a significant predictor for survival in younger patients, those under age 65 (*P* = 0.007, HR = 1.73). For a Kaplan-Meier plot, the median expression value was chosen as a non-biased cut point for dichotomizing the population. Higher levels were associated with poorer survival with *P* = 0.01 and HR = 2.00 (Fig. 6). When examining men and women in the younger age group separately, AGR2 was predictive for survival in both groups. In a multivariate model with stage, grade, age and AGR2 staining intensity (Table 4), AGR2 was an independent predictor of survival in patients under 65 (*P* = 0.002). However, this trend was not observed in patients over age 65 (*P* = 0.964, HR = 0.993). When dividing all patients by stage only, AGR2 as a continuous variable was predictive for survival in stage I [*P* = 0.047, hazard ratio (HR) = 1.50] but not higher stages: stage II (*P* = 0.13, HR = 1.50); stage III (*P* = 0.25, HR = 1.30); stage IV (*P* = 0.61, HR = 1.33). When examining younger patients with stage I, although hazard ratio was slightly higher (HR = 1.85, the *P*-value did not reach significance because of the smaller size of the patient group. AGR2 did not predict survival differences when patients were divided by histology (adenocarcinoma *vs*. squamous carcinoma) or by smoking status.

**Table 4:** Multivariate cox model for younger patients including AGR2, tumor stage, grade and patient age

Multivariate cox model for patients under age 65		
Variable	Hazard ratio (95 % confidence interval)	*P*-value
AGR2	1.86 (1.25 -2.76)	0.002
Stage	1.70 (1.32 -2.20)	3.89E-05
Grade	1.56 (1.10 -2.23)	0.013
Age	1.005 (0.97- 1.04)	0.796

## Discussion

AGR2 is classified as a member of the protein disulfide isomerase (PDI) family of proteins on the basis of amino acid sequence homology. It is localized to the endoplasmic reticulum (ER) as well as secreted [3]. It has also been found in the nucleus and on the cell surface. PDI enzymes contain CXXC domains, which function in oxidation/reduction reactions and isomerization of disulfide bridges. Several PDI family members including AGR2 contain a CXXS motif, and exhibit a lower enzymatic activity than those with CXXC domains [2]. In normal tissues, AGR2 is strongly expressed in lung, trachea, GI tract, and tissues that contain mucin secreting cells or have endocrine functions [2]. Like the bladder urothelium, AGR2 is expressed in the normal bronchial epithelium. Unlike urothelial carcinoma cells, where AGR2 expression is frequently absent, the lung cancer cells retain AGR2. Like prostate, pancreatic, and breast cancer, tumor expression decreases somewhat with increasing grade. With this common trend, we expected cancer expression of AGR2 to be correlated with better patient survival as was demonstrated for prostate cancer [6]. However, this was not the case for lung cancer, in particular, for patients younger than 65. Instead, like high-grade serous ovarian cancer, higher cancer AGR2 expression is correlated with poorer survival. A possible explanation is that AGR2 functions differently in the cancer cell types, or that other differentially expressed genes affect AGR2 function. Depending on the cancer cell types, AGR2 may play roles in differentiation (as in low grade prostate tumors), cell growth and migration (as in advanced prostate cancer) [13].

AGR2 transcription is reported to be regulated by both estrogen and androgen receptors. Upregulation of AGR2 was seen in response to estradiol [26]. AGR2 was identified as being differentially expressed in estrogen receptor (ERα) positive breast cancer cell lines. Treatment of breast cancer patients with Letrazole resulted in decreased AGR2 transcription [26]. Interestingly, tamoxifen enhanced AGR2 expression [27], and AGR2 was found to be a predictor of poorer prognosis in tamoxifen-treated breast cancers [28]. Binding sites for FOXA1, FOXA2, hepatic nuclear factor 1, NF-κB and SOX9 were identified in the promoter region of *AGR2* [26]. MAPK/ERK signaling was shown to regulate AGR2 expression in tumor cell lines in response to physiological stress from serum and oxygen depletion [29]. Vanderlaag et al. showed AGR2 could regulate cell growth, cell cycle progression, and survival in breast cancer cells through modulation of cyclin D1, ERα and survivin [30]. In addition, AGR2 can negatively regulate the p53 transcriptional pathway [31]. Dong et al. reported that binding of EGFR to AGR2 in the ER was necessary for transport of EGFR to the cell surface for EGFR signaling [32]. Huang et al. suggested various proteins and pathways influenced by AGR2 that may affect tumor metastasis including MAST1, SOAT2, POU2AF1 and IFI6 [33]. These studies demonstrate the multi-faceted functional aspect of this protein. Therefore, depending on the precise interaction in particular cell types AGR2 can have different effects on tumor cell behavior. Further studies on the molecular biology of AGR2 in cancer as well as normal cell types are needed.

Our study results differed in some aspects with those in the literature. In studying AGR2 in lung cancers, Fritzsche et al. [9] found no association of AGR2 expression and survival in 95 cases of NSCLC. Their conclusion was based on a smaller number of patients without the cohort dichotomization along age 65. When we considered our entire NSCLC population, we also did not find a significant predictive value for AGR2. The predictive value became significant only when younger patients were considered. Their paper, as well as that of Pizzi et al. [34], reported stronger staining in adenocarcinomas *vs*. squamous carcinomas, similar to our results. Inoue et al. [35] reported AGR2 expression in almost all adenocarcinomas, 96 % squamous carcinomas and 3/3 large cell carcinomas, as well as AGR2 RNA in positive lymph nodes. This contrasts with the general lower expression in squamous carcinomas and large cell carcinomas in our series. Chung et al. [36] reported that AGR2 was up-regulated almost 10-fold in lung adenocarcinoma, and negative expression (seen in 6 % of cases) was associated with poor survival. We did not observe AGR2 up-regulation in cancer cells *vs*. normal cells. Chung et al. [37], however, also reported a significantly higher mean serum AGR2 level even for stage I lung adenocarcinoma (AUC = 0.858), and positive serum AGR2 to be associated with recurrence after surgery (*P* = 0.025) and poor prognosis. Park et al. showed AGR2 to be present within the ER of intestinal secretory epithelial cells, to interact with MUC2 and to be a necessary requirement for mucus production and protection from colitis [38]. That adenocarcinomas with areas of mucinous histologic patterns in our study had higher AGR2 levels on average fits with a role for AGR2 in mucin production.

AGR2 could potentially become a viable serum biomarker for lung cancer [37]. In healthy people, the serum level of AGR2 is non-detectable or very low. This was borne out by mass spectrometry proteomics [39] and query of the *PeptideAtlas* database [40] that showed very low AGR2 signature peptide counts reported to date.

## Conclusions

Normal lung epithelial cells express AGR2 and most NSCLC cells retain this marker. For NSCLC in younger patients, higher tumor AGR2 expression is correlated with a poorer survival in our cohort. Although not as strong a finding, patients with stage I cancer also showed poorer survival with higher levels of AGR2, which could suggest a deleterious role for AGR2 in lung cancer.

## Abbreviations

AGR2: Anterior gradient 2
AUC: Area under the curve
EGFR: Epidermal growth factor receptor
ER: Endoplasmic reticulum
ERα: Estrogen receptor alpha
FOXA1: FOXA2, forkhead box
G: Tumor grade
GI: Gastrointestinal
HR: Hazard ratio
IFI6: Interferon alpha-inducible protein
MAST1: Microtubule associated serine/threonine kinase
MUC2: Mucin 2
NF-κB: Nuclear factor of kappa light polypeptide gene enhancer in B-cells
NSCLC: Non-small cell lung cancer
PDI: Protein disulfide isomerase
POU2AF1: POU class 2 associating factor
SEM: Standard error of the mean
SOAT2: Sterol O-acyltransferase
SOX9: Sex determining region Y-box
TMA: Tissue microarray
